# Transient receptor potential vanilloid 1 and 4 double knockout leads to increased bone mass in mice

**DOI:** 10.1016/j.bonr.2020.100268

**Published:** 2020-04-23

**Authors:** Haruki Nishimura, Makoto Kawasaki, Manabu Tsukamoto, Kunitaka Menuki, Hitoshi Suzuki, Takanori Matsuura, Kazuhiko Baba, Yasuhito Motojima, Teruaki Fujitani, Hideo Ohnishi, Yoshiaki Yamanaka, Kenji Kosugi, Yasuaki Okada, Kotaro Tokuda, Takafumi Tajima, Toru Yoshioka, Nobukazu Okimoto, Yoichi Ueta, Akinori Sakai

**Affiliations:** a Department of Orthopaedic Surgery, School of Medicine, University of Occupational and Environmental Health, 1-1 Iseigaoka, Yahatanishi-ku, Kitakyushu 807-8555, Japan; b Department of Orthopaedics, Shimura Hospital, 3-13 Funairimachi Naka-ku, Hiroshima 730-0841, Japan; c Okimoto Clinic, 185-4 Yutakamachikubi, Kure, Hiroshima 734-0304, Japan; d Department of Physiology, School of Medicine, University of Occupational and Environmental Health, 1-1 Iseigaoka, Yahatanishi-ku, Kitakyushu 807-8555, Japan

**Keywords:** TRPV, transient receptor potential vanilloid, V1KO, TRPV1 knock out, V4KO, TRPV4 knock out, DKO, double knock out, WT, wild type, PCR, polymerase chain reaction, DXA, dual-energy X-ray absorption, CT, computed tomography, POc, preosteoclast, RANKL, receptor activator of nuclear factor-kappa B ligand, RANK, receptor activator of nuclear factor-kappa B, BMD, bone mineral density, TRACP, tartrate-resistant acid phosphatase, ALP, alkaline phosphatase, MNCs, multinucleated cells, CB, cannabinoid, BMSCs, bone marrow mesenchymal stem cells, Transient receptor potential vanilloid, Micro-CT, Bone histomorphometry, Cell culture, Osteogenesis, Preosteoclast

## Abstract

Calcium balance is important in bone homeostasis. The transient receptor potential vanilloid (TRPV) channel is a nonselective cation channel permeable to calcium and is activated by various physiological and pharmacological stimuli. TRPV1 and TRPV4, in particular, have important roles in intracellular Ca^2+^ signaling and extracellular calcium homeostasis in bone cells. TRPV1 and TRPV4 separately mediate osteoclast and osteoblast differentiation, and deficiency in any of these channels leads to increased bone mass. However, it remains unknown whether bone mass increases in the absence of both TRPV1 and TRPV4. In this study, we used *TRPV1* and *TRPV4* double knockout (DKO) mice to evaluate their bone mass *in vivo*, and osteoclast and osteoblast differentiation *in vitro*. Our results showed that DKO mice and wild type (WT) mice had no significant difference in body weight and femur length. However, the results of dual-energy X-ray absorption, microcomputed tomography, and bone histomorphometry clearly showed that DKO mice had higher bone mass than WT mice. Furthermore, DKO mice had less multinucleated osteoclasts and had lower bone resorption. In addition, the results of cell culture using flushed bone marrow from mouse femurs and tibias showed that osteoclast differentiation was suppressed, whereas osteoblast differentiation was promoted in DKO mice. In conclusion, our results suggest that the increase in bone mass in DKO mice was induced not only by the suppression of osteoclast differentiation and activity but also by the augmentation of osteoblast differentiation and activity. Our findings reveal that both the single deficiency of TRPVs and the concurrent deficiency of TRPVs result in an increase in bone mass. Furthermore, our data showed that DKO mice and single KO mice had varying approaches to osteoclast and osteoblast differentiation *in vitro*, and therefore, it is important to conduct further studies on TRPVs regarding the increase in bone mass to explore not only individual but also a combination of TRPVs.

## Highlights

• Knockout of either *TRPV1* or *TRPV4* results in increased bone mass in mice.
• This study evaluates the effects of *TRPV1* and *TRPV4* double knockout (DKO) in mice.
• Concurrent *TRPV1* and *TRPV4* deficiency increases mouse bone mass.
• *TRPV1* and *TRPV4* DKO suppresses osteoclast differentiation and activity.
• *TRPV1* and *TRPV4* DKO enhances osteoblast differentiation and activity.

## 1 Introduction

The skeletal system is affected by both intracellular Ca^2+^ signaling and external Ca^2+^ balance in bone cells. Many functions, including sensing of osmotic and mechanical stimuli, signal transduction, and differentiation of osteoblasts, osteoclasts, and chondrocytes, are regulated by intracellular Ca^2+^ signaling. Therefore, maintaining healthy levels of intracellular Ca^2+^ is essential for bone homeostasis. As such, defects in transporters involved in Ca^2+^ signaling are believed to influence bone structure and function (Blair et al., 2007).

Transient receptor potential (TRP) channels are transmembrane protein channels composed of six transmembrane segments and are present in numerous organisms (Venkatachalam and Montell, 2007; Montell and Rubin, 1989). The structure of this channel system resembles that of voltage-gated cation channels, but it has a largely different composition of the positively charged amino acid residues that determine voltage sensing (Morita et al., 2007). TRP channels can be further classified into seven subfamilies: TRP canonical (TRPC), TRP melastatin (TRPM), TRP vanilloid (TRPV), TRP mucolipin (TRPML), TRP polycystin (TRPP), TRP ankyrin (TRPA), and TRP NO-mechanoreceptor potential (NOMP)-like (TRPN) (Nilius and Owsianik, 2011). TRP channels play important roles in a large variety of physiological functions, ranging from sensation (pheromone signaling; visual, auditory, and taste transduction; nociception; and temperature sensation) to motility (muscle contraction and vasomotor control). Furthermore, TRP channels control body fluid balance, blood circulation, mineral absorption, gut motility, bladder and airway hypersensitivities, cell growth, and survival (Nilius et al., 2007). TRP channels also play a crucial role in bone homeostasis by modulating intracellular Ca^2+^ signaling and extracellular calcium balance in bone cells (Lieben and Carmeliet, 2012).

The TRPV family contains six mammalian members, TRPV1–TRPV6. TRPV1 is present on osteoclasts and osteoblasts and accelerates the differentiation of both cell types (Lieben and Carmeliet, 2012). The nuclear factor of activated T cells 1 gene (*NFATc1*) is essential for osteoclast differentiation, and the knockout of *TRPV1* results in decreased osteoclastogenesis *via* the suppression of *NFATc1* expression and activation (He et al., 2017). In addition, a previous study reported that *TRPV1* knockout (V1KO) mice had higher bone mass than did wild type mice (Rossi et al., 2014). On the other hand, TRPV4 is present on osteoclasts, osteoblasts, and chondrocytes. TRPV4 is located on the basolateral membrane of osteoclasts. It regulates intracellular Ca^2+^ signaling and participates in osteoclast differentiation. TRPV4 regulates the steady Ca^2+^ influx at the late stage of osteoclast differentiation, which is essential for NFATc1-controlled gene transcription, and it modulates osteoclast terminal differentiation and resorptive capacity. A previous study reported that *TRPV4* knockout (V4KO) osteoclasts had lower intracellular Ca^2+^ levels, NFATc1 activity, osteoclast differentiation, and resorptive capacity than did wild type osteoclasts (Masuyama et al., 2012). *In vivo*, V4KO mice had higher bone mass, but lower osteoclast abundance and bone resorption than did wild type mice (Masuyama et al., 2008).

Evidently, both TRPV1 and TRPV4 modulate osteoclast differentiation, and impairing these channels increases bone mass. However, it remains unknown whether bone mass increases with simultaneous deficiencies in TRPV1 and TRPV4. We hypothesized that simultaneous deficiencies of TRPV1 and TRPV4 potently suppress osteoclast differentiation, which may result in a significant increase in bone mass. In this study, we used *TRPV1* and *TRPV4* double knockout (DKO) mice to evaluate the effects of DKO on mouse bone mass *in vivo* and on osteoclast and osteoblast differentiation *in vitro*. Consequently, our results show that DKO mice have a higher bone mass than wild type mice due to not only the suppression of osteoclast differentiation but also the exaggeration of osteoblast differentiation. Taking this result into consideration, the concurrent deficiency of *TRPV1* and *TRPV4* is probably more effective for an increase in bone mass than individual deficiency. Our findings may contribute to the further investigation of the mechanism and a new treatment of osteoporosis associated with TRPV1 and TRPV4.

## 2 Materials and methods

### 2.1 Experimental animals

**Supplemental Fig. S1:**
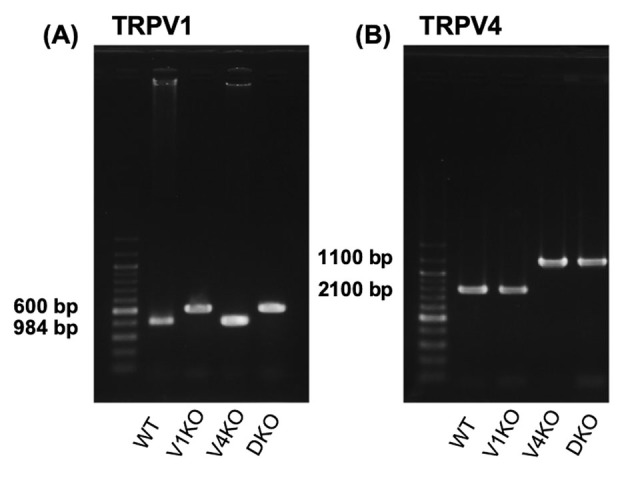
Polymerase chain reaction (PCR) for *TRPV1* and *TRPV4* with C57BL/6J (WT), TRPV1 knockout (V1KO), TRPV4 knockout (V4KO), and TRPV1 and 4 double knockout (DKO) mice. (A) The result of PCR for *TRPV1* is shown. The single band at 600 bp represents the genetic deletion of *TPPV1*. (B) The result of PCR for *TRPV4* is shown. The single band at 1100 bp represents the genetic deletion of *TPPV4*.

Adult male C57BL/6J (wild type: WT) mice, V1KO mice, V4KO mice, and DKO mice (10 weeks of age, weighing 23.0–29.1 g) were used for all experiments (*n* = 4–8 per group). WT mice were purchased from Charles River Japan (Tokyo, Japan). V1KO mice were a kind gift from Dr. D. Julius (University of California-San Francisco), and V4KO mice were a kind gift from Dr. M. Imai (Jichi Medical University, Tochigi, Japan). After receiving permission from the Animal Experiment Committee and Gene Recombination Experiment Safety Committee, DKO mice were generated by mating TRPV1−/− with TRPV4−/− mice. Genetic ablation of both TRPV1 and TRPV4 in the F1 DKO mouse was confirmed by a polymerase chain reaction of genomic DNA extracted from the mouse tail. The DKO mice used in this study (F2) were generated from F1 DKO adult male and female mice. The result of PCR for TRPV1 and TRPV4 in WT, V1KO, V4KO, and DKO mice is shown in Supplemental Fig. S1. All mice used in the present study were housed and kept in our animal research center as described previously (Motojima et al., 2018), and all procedures were performed in accordance with the guidelines on the use and care of laboratory animals established by the Physiological Society of Japan and under the regulation of the Ethics Committee of Animal Care and Experimentation of the University of Occupational and Environmental Health of Japan.

### 2.2 Evaluation of body weight, bone size, and bone mineral density in WT and DKO mice

The body weight of both WT and DKO mice (10 weeks of age) was measured with a digital weighing scale. The lengths of the femurs were measured as previously described (Watanuki et al., 2002). The distance from the top of the greater trochanter to the distal end of the lateral femoral condyle was measured with a digital caliper (Digimatic Caliper; Top Man, Hyogo, Japan). In addition, the bone mineral density (BMD) of the right femurs was evaluated by dual-energy X-ray absorptiometry (DXA; DCS-600, Aloka, Tokyo, Japan).

### 2.3 Evaluation of bone microstructure in WT and DKO mice by micro-CT

The trabecular bone and cortical bone in the right femur of WT and DKO mice were evaluated through microcomputed tomography (micro-CT), as previously described (Tsukamoto et al., 2019a, Tsukamoto et al., 2019b). A micro-CT system (CosmoScan GX; Rigaku, Tokyo, Japan) was used, and the minimum threshold for bone was 304 mg/cm^3^. The bone microstructure parameters were evaluated with Analyze 12.0 software (AnalyzeDirect, Inc., KS, USA) and presented as follows: trabecular bone volume (BV/TV: %), trabecular number (Tb.N: 1/mm), trabecular thickness (Tb.Th: mm), trabecular separation (Tb.Sp: mm), connectivity density (Conn.D: 1/mm3), structure model index (SMI), total cross-sectional area inside the periosteal envelope (Tt.Ar: mm2), cortical bone area (Ct.Ar: mm^2^), cortical area fraction (Ct.Ar/Tt.Ar: %), and average cortical thickness (Ct.Th: mm). Parameters are based on published guidelines (Bouxsein et al., 2010).

### 2.4 Bone histomorphometry in WT and DKO mice

Bone histomorphometric analysis was performed as described previously (Tsukamoto et al., 2016). For this analysis, bone labeling was performed by subcutaneous injection of calcein (20 mg/kg body weight) seven and three days before sacrifice. Coronal sections (5-μm thick) of the left proximal tibial specimens embedded in methyl methacrylate after Villanueva's bone staining were used to evaluate BV/TV (%), osteoid surface (OS/BS: %), osteoblast surface (Ob.S/BS: %), osteoclast surface (Oc.S/BS: %), eroded surface (ES/BS, %), mineralizing surface (MS/BS: %), mineral apposition rate (MAR: μm/day), and surface referent bone formation rate (BFR/BS: mm^3^/mm^2^/year). In addition, 5-μm thick coronal sections of the right proximal tibial specimens were embedded in paraffin after tartrate-resistant acid phosphatase (TRACP) staining (TRACP & ALP Double-stain Kit; TaKaRa, Shiga, Japan) and were used to evaluate osteoclast number (Oc.N/BS: N/mm). The number of preosteoclasts (POc.N/BS: N/mm) or TRACP-positive mononucleated cells (Xie et al., 2014), was also measured. The cells that were stained by TRACP and contained two or more nuclei were identified as osteoclasts (Tsukamoto et al., 2016). The abbreviations for the histomorphometric parameters were used in accordance with the recommendations of the Histomorphometry Nomenclature Committee of the American Society for Bone and Mineral Research (Parfitt et al., 1987; Dempster et al., 2013).

### 2.5 Cell cultures from WT, V1KO, V4KO, and DKO mice

Cell culture for alkaline phosphatase (ALP)-positive colony-forming unit-fibroblastic (CFU-f) cells and osteoclast-like TRACP-positive multinucleated cells (MNCs) was performed as previously described with minor changes (Tsukamoto et al., 2016).

#### 2.5.1 ALP-positive CFU-f

The bilateral femurs and tibias were flushed with 5 ml of α-Minimum Essential Medium (α-MEM, Nacalai Tesque, Kyoto, Japan), and bone marrows were obtained. For determination of the ALP-positive CFU-f colony formation, 1 × 10^5^ marrow cells/well were plated on 6-well plates (Thermo Fisher Scientific, Roskilde, Denmark). On day 10, the CFU-f colonies were fixed and stained with ALP (TRACP & ALP Double-stain Kit). Colonies containing at least 50 cells were defined as CFU-f. The total number of CFU-f colonies and the number of ALP-positive CFU-f colonies were counted under a light microscope (Watanuki et al., 2002; Sakata et al., 1999; Sakai et al., 2002; Okuma et al., 2017).

#### 2.5.2 TRACP-positive MNCs

Bone marrows were obtained using the same method described in Section 2.5.1. For the determination of the TRACP-positive MNCs, 1.5 × 10^6^ marrow cells/well were plated on 24-well plates (Thermo Fisher Scientific). On day 7, the cultures were fixed and stained with TRACP (TRACP & ALP Double-stain Kit). The number of TRACP-positive MNCs that had three or more nuclei, and the number of TRACP-positive mononucleated cells were counted under a light microscope (Sakata et al., 1999; Sakai et al., 1996, Sakai et al., 1999; Lee et al., 2006).

### 2.6 Real-time PCR

#### 2.6.1 RNA isolation and first-strand cDNA synthesis

RNA isolation was performed as previously described (Tsukamoto et al., 2016, Tsukamoto et al., 2019a; Tajima et al., 2018). First strand cDNA was reverse-transcribed from total RNA (1 μg) using Moloney murine leukemia reverse transcriptase (SuperScript II; Life Technologies, Rockville, MD) and oligo(dT) 12–18 primers (Life Technologies).

#### 2.6.2 Quantitative real-time PCR

Quantitative real-time PCR (RT-PCR) analysis was performed as previously described (Tsukamoto et al., 2016, Tsukamoto et al., 2019a; Tajima et al., 2018) using an iCycler apparatus (Bio-Rad Laboratories, Hercules, CA, USA) with iCycler Optical System Software version 3.1 (Bio-Rad). The quantitative PCR reactions for Runx2, osterix, and osteocalcin were performed in 10-μl reactions containing 5 ng of cDNA, 0.5 pM primers, and 5 μl of iQ SYBR Green Supermix (Bio-Rad). The primers used in this study were designed using Primer3 software and synthesized by Sigma-Aldrich Japan K.K. Genosys Division (Hokkaido, Japan). β-Actin served as an internal control. The amplification conditions were an initial 3 min at 95 °C and 40–50 cycles of denaturation at 95 °C for 30 s, annealing at 65 °C for 30 s, and extension at 72 °C for 30 s. The mRNA expression levels were normalized with β-actin mRNA expression levels and expressed as relative values (fold change) to the expression levels in WT mice.

### 2.7 Statistical analysis

All results are shown as the mean ± standard error of the mean (SEM). Student's *t*-test was used to detect differences between the WT and DKO mice. One-way ANOVA was used for multiple comparisons between WT, V1KO, V4KO, and DKO mice in the cell culture and RT-PCR analysis. Statistically significant differences were defined as *p* < 0.05. All statistical analyses were performed with STATA/IC 14 (StataCorp, College Station, TX, USA).

## 3 Results

### 3.1 Body weight, bone size, and examination of bone mineral density

**Fig. 1:**
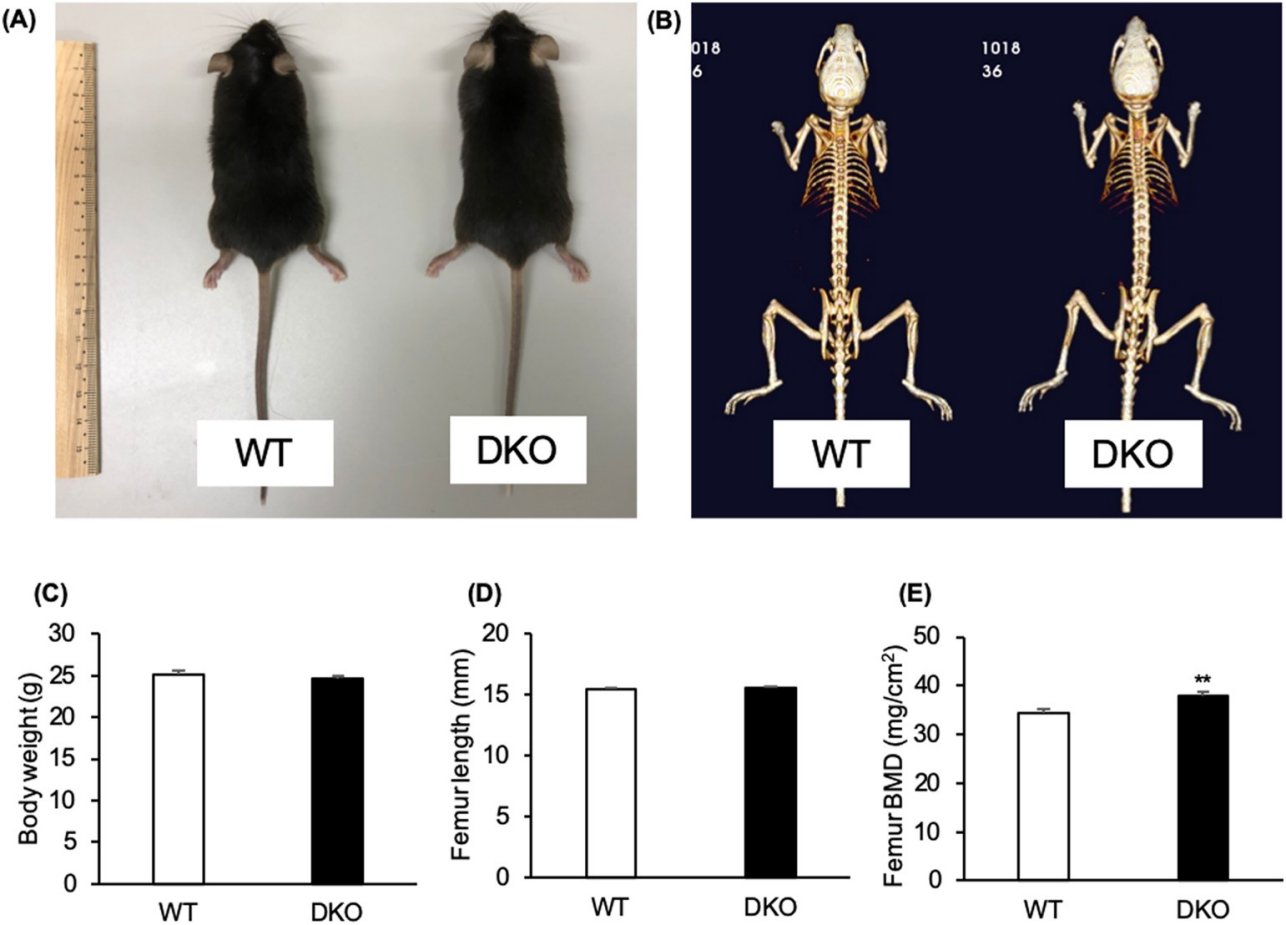
The physique, body weight, femur length, and bone mineral density (BMD) of the whole femur of C57BL/6J (WT) and transient receptor vanilloid 1 and 4 (*TRPV1* and *TRPV4*) double knockout (DKO) mice. (A) The gross appearances and (B) three-dimensional images of the whole-body skeletons of WT and DKO mice are shown. The mean values of (C) body weight measured by a digital scale, (D) femur length measured by a digital caliper, and (E) BMD of the whole femur analyzed by DXA of WT and DKO mice are shown (n = 8 in each group). The data are presented as means ± SEM. Student's *t*-test, **p < 0.01 compared to WT.

The WT and the DKO mice had no remarkable difference in both gross appearance and the three-dimensional whole-body skeleton image (Fig. 1A and B). Likewise, no remarkable difference in body weight and femur length (Fig. 1C and D) was observed between the WT and DKO mice. However, the DKO mice had remarkably higher BMD in femurs than the WT mice as analyzed by DXA (Fig. 1E).

### 3.2 Evaluation of the bone microstructure with micro-CT

**Fig. 2:**
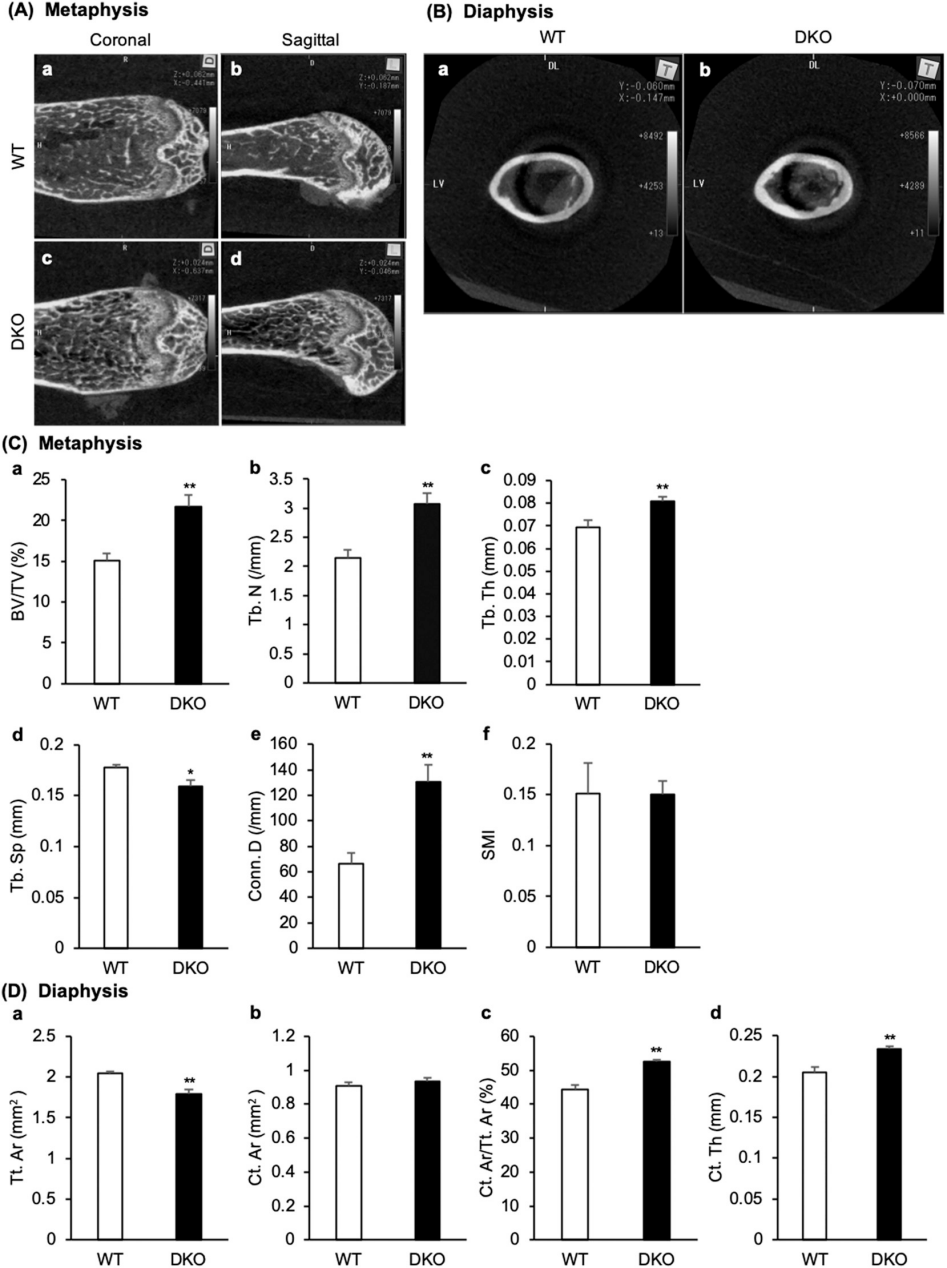
Micro-computed tomography (micro-CT) evaluation of the metaphysis and diaphysis of the femur for C57BL/6J (WT) and transient receptor vanilloid 1 and 4 (*TRPV1* and *TRPV4*) double knockout (DKO) mice. (A) Micro-CT images of the metaphysis of the femur of WT (a, c) and DKO (b, d) mice are shown. (B) Micro-CT images of the diaphysis of the femur of WT (a) and DKO (b) mice are shown. (C) The mean values of BV/TV (a), Tb. N (b), Tb. Th (c), Tb. Sp (d), Conn. D (e), and SMI (f) of the metaphysis of the femur are shown. (D) The mean values of Tr. Ar (a), Ct. Ar (b), Ct. Ar/Tt. Ar (c), and Ct. Th (d) of the diaphysis of the femur are shown. (n = 8 in each group). The data are presented as means ± SEM. Student's *t*-test, *p < 0.05 and **p < 0.01 compared to WT. TRPV: transient receptor potential vanilloid.

Micro-CT images showed remarkable differences in the trabecular bone in the femoral metaphysis and in the cortical bone in the femoral diaphysis between the WT and the DKO mice (Fig. 2A and B). Specifically, the DKO mice had remarkably higher BV/TV, Tb. N, Tb. Th, Conn. D of the trabecular bone in the femoral diaphysis than the WT mice. On the other hand, the Tb. Sp of the trabecular bone in the femoral diaphysis in DKO mice was remarkably lower than that of the WT mice (Fig. 2C). With respect to cortical bone at the femur diaphysis, the Tt. Ar in the DKO mice was significantly lower, and the Ct. Ar/Tt Ar and Ct. Th in the DKO mice were remarkably higher than those in the WT mice (Fig. 2D).

### 3.3 Bone histomorphometry

**Fig. 3:**
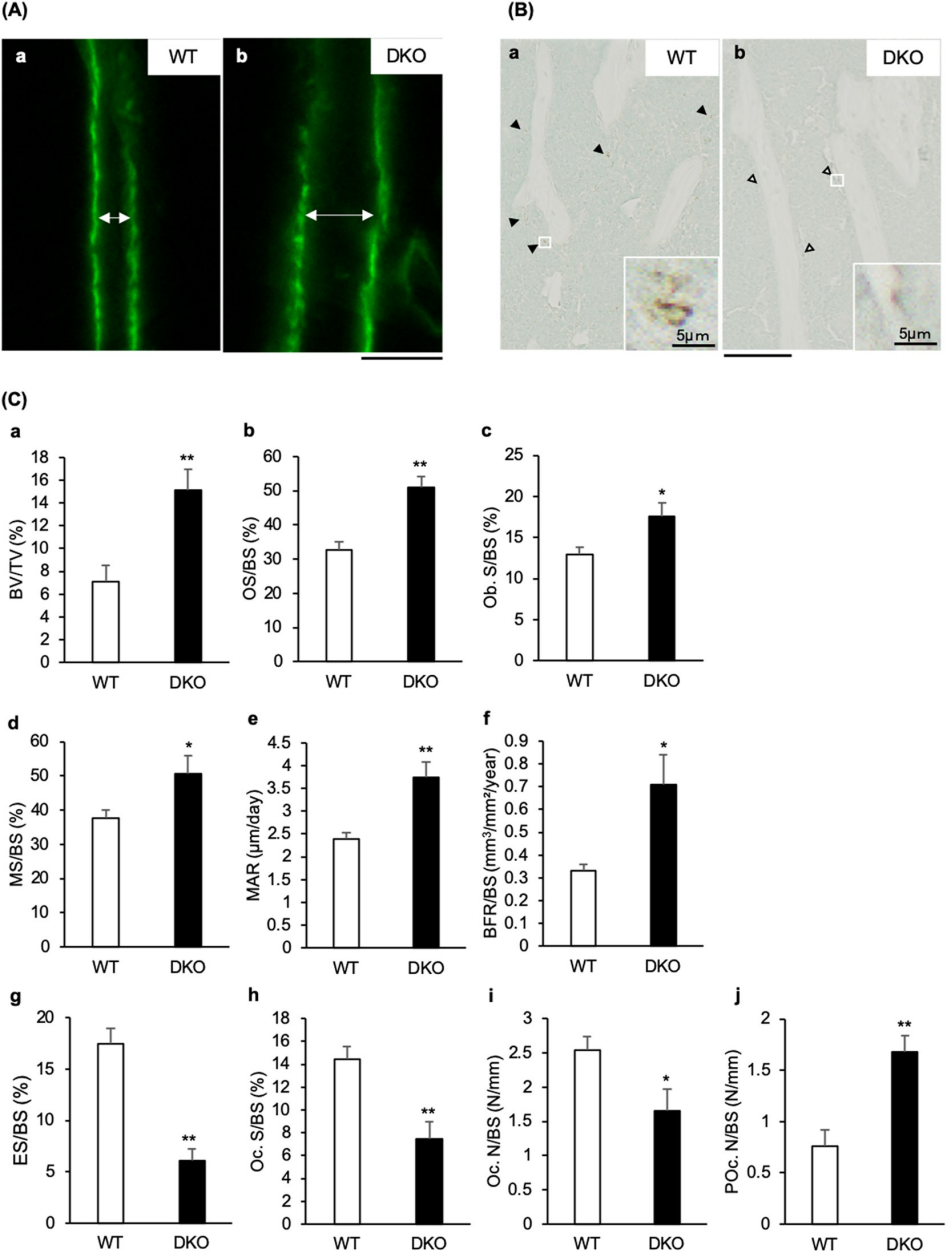
Trabecular bone volume, formation, and resorption evaluated by bone histomorphometry of the proximal tibias of C57BL/6J (WT) and transient receptor vanilloid 1 and 4 (*TRPV1* and *TRPV4*) double knockout (DKO) mice. (A) Calcein double-labeling fluorescent microscopic images of the trabecular bone in the proximal tibias of WT (a) and DKO (b) mice are shown (scale bar = 10 μm). (B) The microscopic images of the proximal tibias with tartrate-resistant acid phosphatase (TRACP) staining using non-decalcified specimens of WT (a) and DKO (b) are shown (scale bar = 500 μm). Filled black triangles indicate TRACP-positive cells in WT mice, and black-outlined triangles indicate TRACP-positive cells in DKO mice. Magnified images of typical TRACP-positive cells for WT and DKO mice within the white squares are shown at the lower right (scale bar = 5 μm). (C) The mean values of BV/TV (a), OS/BS (b), Ob. S/BS (c), MS/BS (d), MAR (e), BFR/BS (f), ES/BS (g), Oc. S/BS (h), Oc. N/BS (i), and POc. N/BS (j) of the trabecular bone in the proximal tibias of WT and DKO mice are shown. (n = 5 in each group). The data are presented as means ± SEM. Student's *t*-test, *p < 0.05 and **p < 0.01 compared to WT.

The results of bone histomorphometry showed that BV/TV, OS/BS, Ob.S/BS, and MS/BS in the DKO mice were remarkably higher than those in the WT mice (Fig. 3C-a-d). Under the parameters of trabecular bone formation, MAR and BFR/BS in the DKO mice were remarkably higher than those in the WT mice (Fig. 3C-e and f). On the other hand, ES/BS in the DKO mice was remarkably lower than that in the WT mice (Fig. 3C-g). Further, under the parameters of trabecular bone resorption, Oc. S/BS and Oc. N/BS in the DKO mice were remarkably lower, while POc. N/BS in the DKO mice was remarkably higher than those in the WT mice (Fig. 3C-h, i, and j).

### 3.4 Cell culture

**Fig. 4:**
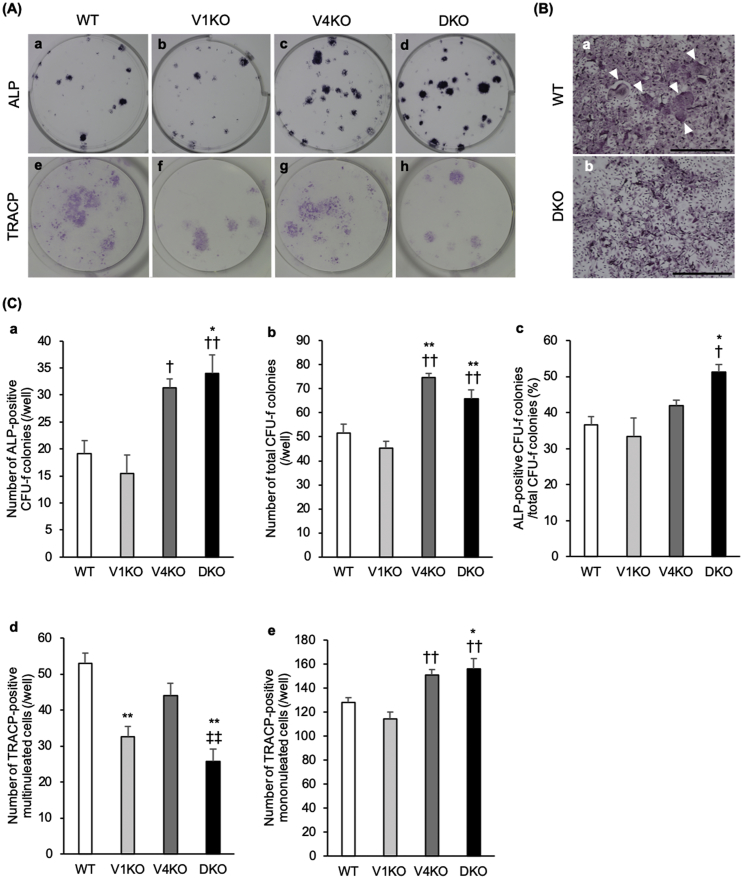
Evaluation of osteoblast and osteoclast differentiation. Cell culture assay was performed using bone marrow cells flushed from the bilateral femurs and tibias of C57BL/6J (WT), transient receptor vanilloid 1 (*TRPV1*) knockout (V1KO), *TRPV4* knockout (V4KO), and *TRPV1* and *TRPV4* double knockout (DKO) mice. (A) The images of wells with alkaline phosphatase (ALP)-stained or tartrate-resistant acid phosphatase (TRACP)-stained cultured cells from WT (a, e), V1KO (b, f), V4KO (c, g), and DKO (d, h) mice are shown. (B) The microscopic images of TRACP-stained cultured cells for WT (a) and DKO (b) mice. White arrows indicate multinucleated cells (scale bar = 500 μm). (C) The numbers of ALP-positive CFU-f colonies (a) and total CFU-f colonies (b) per well for each mouse strain and the percentage of ALP-positive CFU-f colonies/total CFU-f colonies (c) for each mouse strain are shown. TRACP-positive multinucleated cells (d), and TRACP-positive mononucleated cells (e) per well for each mouse strain are shown (n = 4 in each group). The data are presented as mean ± SEM. One-way ANOVA, *p < 0.05 and **p < 0.01 compared to WT; †*p* < 0.05 and ††*p* < 0.01 compared to V1KO; ‡‡*p* < 0.01 compared to V4KO.

A primary cell culture using bone marrow cells was prepared to evaluate the ALP-positive CFU-f colonies, total CFU-f colonies, TRACP-positive MNCs, and TRACP-positive mononucleated cells in the WT, V1KO, V4KO, and DKO mice. The number of ALP-positive CFU-f colonies in the V4KO and DKO mice was remarkably higher than that in the V1KO mice, and the number of ALP-positive CFU-f colonies in the DKO mice was remarkably higher than that in the WT mice. However, there was no remarkable difference between the WT and V1KO mice (Fig. 4C-a). The percentage of ALP-positive CFU-f colonies relative to the total CFU-f colonies in the DKO mice was remarkably higher than that in the WT and V1KO mice (Fig. 4C-c). The numbers of TRACP-positive MNCs in the V1KO and DKO mice were remarkably lower than those in the WT mice. In addition, the number of TRACP-positive MNCs in the DKO mice was also remarkably lower than that in the V4KO mice. However, there was no remarkable difference between the WT and V4KO mice (Fig. 4C-d). On the other hand, the numbers of TRACP-positive mononucleated cells in the V4KO and DKO mice were remarkably higher than those in the V1KO mice. The number of TRACP-positive mononucleated cells in the DKO mice was also remarkably higher than that in the WT mice. However, there was no remarkable difference between the WT and V1KO mice (Fig. 4C-e)

### 3.5 The expression of Runx2, osterix, and osteocalcin mRNA

**Fig. 5:**
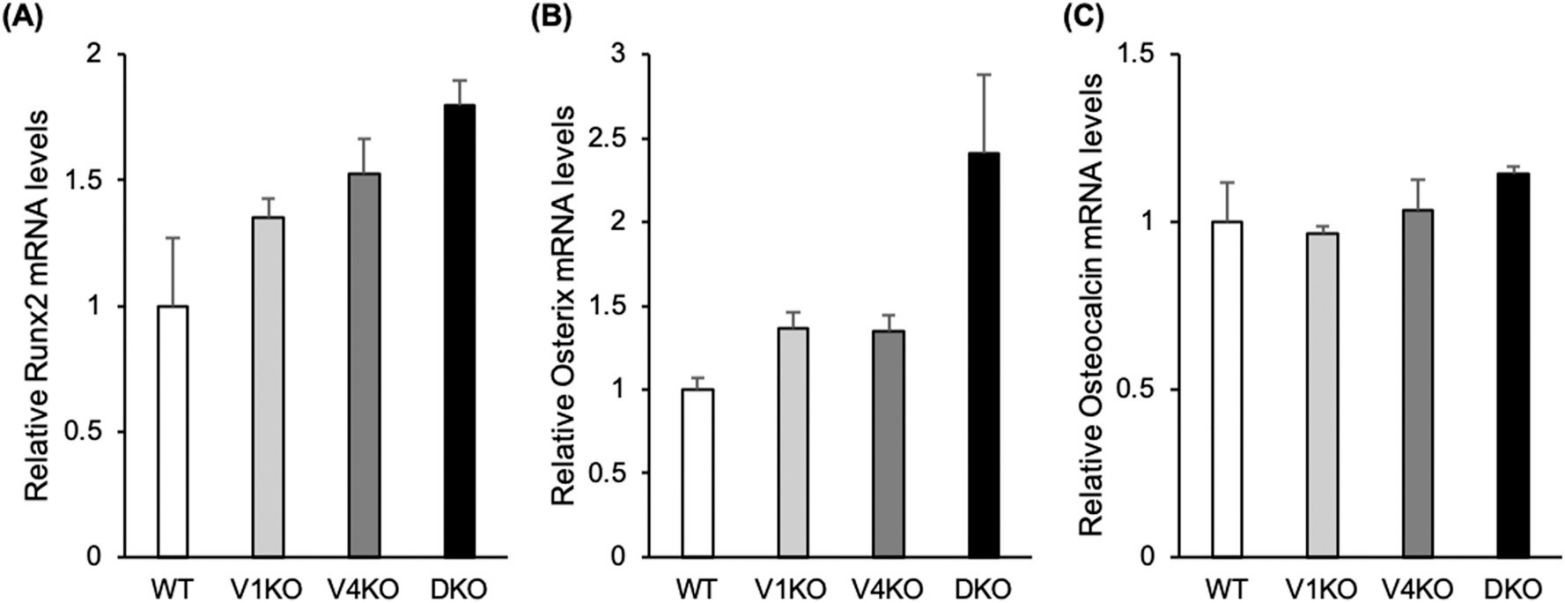
mRNA expression levels of *Runx2*, *osterix*, and *osteocalcin*. Quantitative real-time PCR analysis was performed using bone marrow cells flushed from the bilateral femurs and tibias of C57BL/6J (WT), transient receptor vanilloid 1 (*TRPV1*) knockout (V1KO), *TRPV4* knockout (V4KO), and *TRPV1* and *TRPV4* double knockout (DKO) mice. The mRNA levels of *Runx2* (A), *osterix* (B), and *osteocalcin* (C) for each mouse strain are shown. (n = 4 in each group). The data are presented as mean ± SEM. One-way ANOVA.

Although DKO mice showed the highest mean values of mRNA expression levels of Runx2, osterix, and osteocalcin, there was no statistically significant difference regarding the expression levels of these genes among any groups. However, osterix mRNA expression level of DKO mice tended to be higher than that of WT mice (P = 0.075) (Fig. 5).

## 4 Discussion

The results of DXA, micro-CT, and bone morphometry clearly showed that DKO mice had higher bone mass and had higher BMD, BV/TV, Tb N, and OS/BS. These results corroborate with previous reports on the effects of knockouts of either *TRPV1* or *TRPV4* on bone mass (He et al., 2017; Rossi et al., 2014; Masuyama et al., 2008; van der Eerden et al., 2013). Likewise, a previous study also reported higher Tb N and Tb. Th, and lower Tb. Sp in V1KO mice than in WT (He et al., 2017). Higher body weights have been reported for 10-week old V4KO mice than for WT (O'Conor et al., 2013). We also observed this in the V4KO mice used in this study (data not shown). However, there was no remarkable change in body weight in the DKO mice. Further studies are needed to understand the mechanisms underlying these results. Additionally, there was no change in the femur length of the DKO mice.

The results of bone morphometry and cell culture in this study indicated that bone resorption and osteoclast differentiation in DKO mice were lower than those in WT mice, as shown by lower ES/BS, Oc. S/BS, Oc. N/BS, and TRACP-positive MNCs. Furthermore, the results of bone morphometry and cell culture also suggest that bone formation and osteoblast differentiation were enhanced in DKO mice than in WT mice, as indicated by increased Ob. S/BS, MAR, BFR/BS, number of ALP-positive CFU-f colonies, and percentage of ALP-positive CFU-f colonies/ total CFU-f colonies. These results suggest that osteoclast activity and differentiation were suppressed by *TRPV1* and *TRPV4* deficiency and that the appearance of premature osteoclasts was enhanced. To confirm which transcriptional factors are associated with the increase in osteoblast differentiation in DKO mice, we performed RT-PCR analysis to compare the gene expression of osteoblast-specific genes such as Runx2, osterix, and osteocalcin. Although there was no significant difference in the expression of these genes among any groups probably due to the small number of experimental animals (n = 4), DKO mice showed the highest mean values of the expression of these genes, and osterix mRNA expression level of DKO mice tended to be higher than that of WT mice. Therefore, this result suggested that osterix might play an important role in the increase in osteoblast differentiation in DKO mice.

Idris et al. (2010) have reported that *TRPV1* blockade inhibits osteoclast differentiation *in vitro*, and the administration of TRPV1 antagonist inhibited ovariectomy (OVX)-induced bone loss by reducing the increase in bone resorption *in vivo* and osteoclast and osteoblast differentiation *in vitro*. Rossi et al. (2014) have also reported that the knockout of TRPV1 as well as pharmacological blockade of TRPV1 signaling significantly reduces osteoclast differentiation and activity *in vitro,* and prevents OVX-induced bone loss induced *in vivo*, whereas the expression of cannabinoid (CB) 2 receptors was enhanced, suggesting a possible cross-talk between TRPV1 and CB2 receptors. The stimulation of CB2 receptors significantly reduces the number of active multinucleated osteoclasts *in vitro*. TRPV1 is expressed not only in osteoclasts but also in osteoblasts, and it accelerates differentiation in both cell types; therefore, *TRPV1* blockade inhibits these differentiations (Lieben and Carmeliet, 2012).

Pan et al. (2013) reported that osteoblast apoptosis induced by sodium nitroprusside was largely inhibited by *TRPV1* blockade. Although a specific TRPV1 antagonist could not inhibit osteoblast apoptosis completely, ruthenium red and La^3+^, a non-selective antagonist of TRP, could. Therefore, the author suggested that not only *TRPV1* but also *TRPV4* can mediate osteoblast apoptosis because TRPV4 was also functionally expressed on osteoblasts. TRPV4 regulates a steady Ca^2+^ influx in the late stages of osteoclast differentiation and modulates the terminal differentiation and resorptive capacity of osteoclasts (Masuyama et al., 2008; Negishi-Koga and Takayanagi, 2009). In addition, local acidosis affects the last phase of preosteoclast differentiation and accelerates osteoclast formation. A previous study has reported that a TRPV4-specific agonist partially inhibits acidosis-promoted TRACP-positive multinuclear cell formation (Kato and Morita, 2011). Masuyama et al. (2008) reported that the differentiation and resorptive capacity of V4KO osteoclasts were suppressed *in vitro*. *In vivo*, the bone mass increased, while osteoclast abundance and bone resorption were reduced in V4KO mice. Masuyama et al. (2012) also reported that selective TRPV4 activation in osteoclasts increased its differentiation and resulted in bone loss. Further, TRPV4 modulates osteoclast fusion and migration *via* Ca^2+^/calmodulin-mediated effects on myosin IIa. Mizoguchi et al. (2008) reported that V4KO mice were resistant to bone loss induced by unloading. van der Eerden et al. (2013) reported that male V4KO mice showed lower osteoclast activity and numbers *in vitro*. On the other hand, osteoblast differentiation was enhanced in V4KO mice. Further, the trabecular and cortical bone masses in male V4KO mice were about 20% higher than those in WT mice. Although osteoblast differentiation and activity should be reduced with the suppression of osteoclast differentiation and activity due to the osteoclast-osteoblast coupling system, this effect was not observed in their study *in vivo*. Therefore, they concluded that TRPV4 affects mesenchymal stem cells and limit their differentiation into osteoblasts.

Son et al. (2018) reported that applying a TRPV4 antagonist reduced hypo-osmotic stress-induced significant increases in receptor activator of nuclear factor-kappa B ligand (RANKL) mRNA gene expression and intracellular Ca^2+^ concentration in osteoblasts in mice. RANKL affects osteoclast precursor cells and regulates their differentiation into multinucleated osteoclasts. Therefore, TRPV4 has a major role in RANKL expression. RANKL associates with receptor activator of nuclear factor-kappa B (RANK) on osteoclast precursor cells and triggers osteoclastogenesis. Recent studies have suggested that osteocytic RANKL plays a major role in osteoclastogenesis for a period of bone remodeling. Nevertheless, the main function of osteoblastic RANKL is not well understood. Ikebuchi et al. (2018) reported “RANKL reverse signaling”; the vesicular RANK synthesized by maturing osteoclasts binds to membranous RANKL on preosteoblasts and accelerates osteoblast differentiation and bone formation by activating Runx 2. This RANKL reverse signaling may be one of the causes of the increase in bone mass in DKO mice. In this study, the deficiency in TRPV4 suppressed late-stage osteoclast differentiation, leading to lower osteoclast count but increased POc. count in DKO mice, as shown by bone histomorphometry. In addition, the number of TRACP-positive mononucleated cells also increased in the V4KO and DKO mice *in vitro*. These results suggest that expression of membranous RANK was suppressed but expression of vesicular RANK was increased, and RANKL reverse signaling from these maturing osteoclasts induced the activation of osteoblasts, resulting in increased bone mass in DKO mice.

**Fig. 6:**
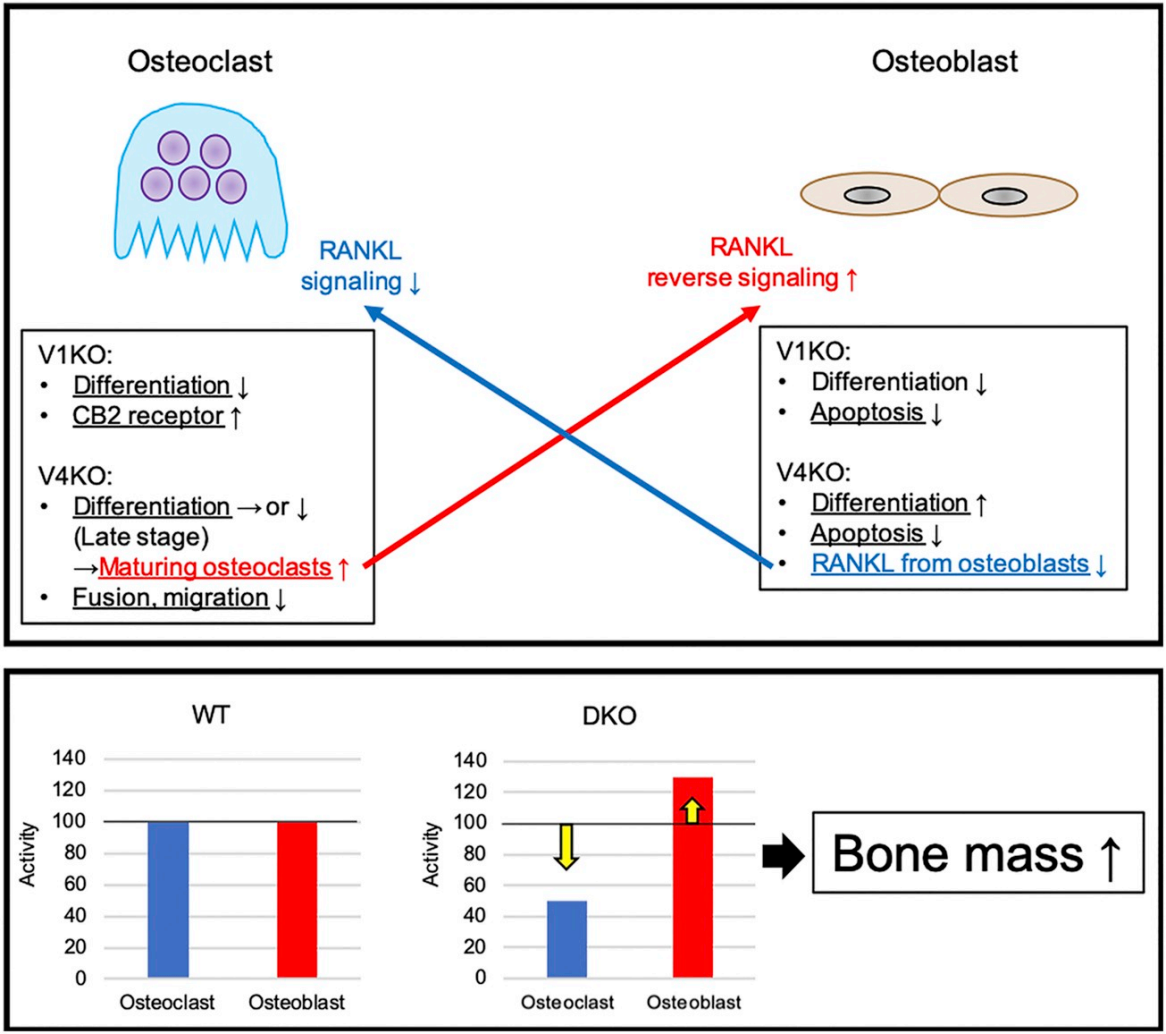
The proposed mechanism for the increase in bone mass in the *TRPV1* and *TRPV4* double knockout (DKO) mice. Transient receptor vanilloid 1 knock out (V1KO), transient receptor vanilloid 4 knock out (V4KO), wild type (WT), cannabinoid (CB), receptor activator of nuclear factor-kappa B ligand (RANKL).

Taken together, we propose that the mechanism of the increase in bone mass in *TRPV1* and *TRPV4* DKO was induced by the following: 1. suppression of osteoclast activity *via* the suppression of osteoclast differentiation, fusion, and migration; and increase in CB2 receptors and decrease in RANKL signaling from osteoblasts; 2. exaggeration of osteoblast activity *via* the promotion of osteoblast differentiation, inhibition of apoptosis, and increase in RANKL reverse signaling from maturing osteoclasts (Fig. 6).

However, there are several limitations to be noted in this study. First, we evaluated and compared bone mass only between WT and DKO mice *in vivo*. We planned to evaluate bone mass *in vivo* among WT, V1KO, V4KO, and DKO mice. However, the V4KO mice had a significantly higher body weight than all other strains of mice, and the V1KO mice had significantly lower body weight than did WT mice (data not shown). It is well known that body weight remarkably impacts bone density (Mazess et al., 1987; Reid et al., 1992; Felson et al., 1993; Marcus et al., 1994; Ravn et al., 1999). Therefore, we hypothesized that bone mass *in vivo* among WT, V1KO, V4KO, and DKO mice might be affected by body weight. However, although there was no significant difference in body weight between DKO and WT mice, DKO mice displayed significantly higher bone mass. For this reason, we regard DKO mice as a unique model in which bone mass increases without body weight being a factor, and therefore, decided to compare only WT and DKO mice *in vivo*. Second, we have not evaluated the bone strength of DKO mice. A previous study reported that the bone material of V4KO mice was less resistant to stress and was less elastic even though it had higher bone mass (van der Eerden et al., 2013). Therefore, the bone strength of DKO mice may be weaker than that of WT mice. In addition, TRPV4 is reported to be essential for flow-induced Ca^2+^ signaling during differentiation in osteoblasts, which indicates that TRPV4 has a critical role in increasing bone mass by mechanical stress (Suzuki et al., 2013). Therefore, further experiments are needed to evaluate the bone homeostasis of DKO mice by applying mechanical stress, such as in a climbing exercise model (Mori et al., 2003). In addition, previous studies reported that genetic anomaly of TRPV4 causes genetic skeletal disorders, such as metatropic dysplasia, brachyolmia, spondylo-epimetaphyseal dysplasia, and parastremmatic dysplasia (Nilius and Owsianik, 2010; Andreucci et al., 2011; Nishimura et al., 2010; Lamandé et al., 2011; Loukin et al., 2011; Graversen et al., 2018). Further, TRPV1 and TRPV4 are present in numerous organs, in which TRPV1 and TRPV4 deficiencies may affect the function of those organs. Although the results of the skeletal evaluation with micro CT did not show any relevant abnormalities in DKO mice, further analysis as to whether DKO mice have any genetic disorders of the skeletal system or organs in detail is also our future task.

In summary, this study has shown that simultaneous deficiencies in *TRPV1* and *TRPV4* result in increased bone mass. Our results suggest that the increase in bone mass in DKO mice was induced not only through the suppression of osteoclast activity but also through the promotion of osteoblast activity. Previous studies have reported that knockout of either *TRPV1* or *TRPV4* results in mice with higher bone mass. To our knowledge, this is the first study to report that simultaneous deficiencies of *TRPV1* and *TRPV4* also cause increased bone mass, and to compare the differentiation of bone cells *in vitro* among simultaneous deficiencies and single deficiency of TRPVs. Although we could not show any advantages of the concurrent deficiency of TRPVs on the increase in bone mass in comparison with the single deficiency of TRPVs, our data revealed that DKO mice and single KO mice have different approaches to osteoclast and osteoblast differentiation. Our results indicate that it is important to explore the mechanisms of TRPVs regarding the increase in bone mass not only individually but also concurrently with a combination of TRPVs.

The following is the supplementary data related to this article.

## Transparency document

Transparency document. Image 1

## CRediT authorship contribution statement

**Haruki Nishimura:**Conceptualization, Methodology, Software, Validation, Formal analysis, Investigation, Data curation, Writing - original draft, Writing - review & editing.**Makoto Kawasaki:**Conceptualization, Validation, Project administration, Funding acquisition.**Manabu Tsukamoto:**Supervision, Conceptualization, Resources, Investigation.**Kunitaka Menuki:**Conceptualization, Funding acquisition.**Hitoshi Suzuki:**Validation, Funding acquisition.**Takanori Matsuura:**Supervision, Conceptualization.**Kazuhiko Baba:**Investigation.**Yasuhito Motojima:**Investigation, Visualization.**Teruaki Fujitani:**Investigation.**Hideo Ohnishi:**Supervision, Conceptualization, Funding acquisition.**Yoshiaki Yamanaka:**Conceptualization, Methodology, Software, Investigation.**Kenji Kosugi:**Software, Validation, Formal analysis, Resources.**Yasuaki Okada:**Methodology, Resources.**Kotaro Tokuda:**Methodology, Resources.**Takafumi Tajima:**Conceptualization, Methodology, Formal analysis, Data curation.**Toru Yoshioka:**Supervision, Conceptualization.**Nobukazu Okimoto:**Supervision, Conceptualization.**Yoichi Ueta:**Conceptualization, Validation, Project administration.**Akinori Sakai:**Conceptualization, Project administration, Funding acquisition.
